# Analysis of YouTube-Based Therapeutic Content for Children with Cerebral Palsy

**DOI:** 10.3390/children11070814

**Published:** 2024-07-02

**Authors:** Yerim Do, Yunjae Oh, Na Young Kim, Juntaek Hong

**Affiliations:** 1 Department and Research Institute of Rehabilitation Medicine, Yonsei University College of Medicine, Seoul 03722, Republic of Korea; doyr62@yuhs.ac; 2 Department of Rehabilitation Therapy, Severance Rehabilitation Hospital, Yonsei University Health System, Seoul 03722, Republic of Korea; oyj93@yuhs.ac; 3 Department of Rehabilitation Medicine, Yongin Severance Hospital, Yonsei University College of Medicine, Yongin 16995, Republic of Korea; kny8452@yuhs.ac

**Keywords:** YouTube, video, cerebral palsy, exercise, neuromotor therapy, quality

## Abstract

Background/Objectives: Cerebral palsy (CP) causes movement and posture challenges due to central nervous system damage, requiring lifelong management. During the COVID-19 pandemic, there was limited access to facility-based treatments, which increased the demand for home-based therapies and digital resources. We analyzed the qualitative and quantitative aspects of YouTube videos focusing on CP therapy for children. Methods: A total of 95 videos were evaluated for content quality using the modified DISCERN (mDISCERN) tool and Global Quality Scale (GQS). The therapeutic program efficacy was assessed via the International Consensus on Therapeutic Exercise and Training (i-CONTENT) tool, Consensus on Therapeutic Exercise Training (CONTENT) scale, and Consensus on Exercise Reporting Template (CERT), and popularity was measured by the video power index (VPI). Results: YouTube-based therapeutic videos for children with CP generally exhibit reliability in video content and effectiveness in therapeutic programming, and no correlations were found between video popularity and quality. However, the qualitative analysis reveals insufficient mention of uncertainty in the treatment principles within the video content as well as a lack of detailed treatment descriptions encompassing aspects such as intensity, frequency, timing, setting, outcome measurement during and post-treatment, and safety considerations within therapeutic programs. In particular, this tendency was consistent regardless of the uploader’s expertise level and the classification of the neuromotor therapy type in contrast to that of the exercise type. Conclusions: YouTube-based content for CP children still has significant limitations in how substantive viewers, such as caregivers, can acquire tailored information and apply practical information to their exercise and treatment programs.

## 1. Introduction

Cerebral palsy (CP) is a lifelong, non-progressive disorder affecting movement and posture due to damage to the central nervous system. Children with CP usually experience musculoskeletal complications, impaired selective motor control, and poor balance, which affect their gross motor function and activities of daily living [1]. Moreover, owing to the nature of CP, which is permanent but changeable depending on variables such as growth, the effective management of complications is essential. Thus, the importance of providing adequate rehabilitation and exercise according to the life cycle cannot be overemphasized [2]. Hence, various modalities have been provided for children with CP. For example, conventional neuromotor techniques, such as neurodevelopmental treatment (Bobath) and the Vojta approach, are widely used in clinical settings to enhance muscle strength, balance, and coordination while reducing stiffness and spasticity [3]. Furthermore, most of the reviewed studies suggest that even a minimal amount of exercise can benefit this population, potentially improving mortality rates [4]. However, with the emergence of the COVID-19 pandemic, access to facility-based treatment became limited. This caused challenges in health delivery systems and increased the demand for home-based programs and related digital information [5,6,7]. Nowadays, people commonly use Internet sources to find and share information. Accordingly, YouTube, a well-known video-sharing site, is utilized to access medical information. Reportedly, 43% of YouTube users use the platform to obtain information about treatment and medical procedures [8]. Furthermore, patients with chronic conditions often depend on Internet-based resources. According to surveys, 75% of these patients were influenced by the knowledge they acquired from the Internet in making decisions about treating their condition. This highlights the potential of YouTube as a platform for sharing health-related information [9].

Despite the practicality of easily obtaining information through this platform, there is a risk of inaccuracy, as anyone can post on this platform, unlike scientific literature that undergoes peer-review processes. In this context, previous studies have conducted a quality assessment of the videos related to various health conditions and have found that approximately 25–30% of these informational videos on YouTube contained misleading information or were of low quality [8,10,11]. Additionally, even in the quality analysis of most videos uploaded by medical professionals, the overall quality appeared to be poor [12]. Considering their potential impact on children with CP, the quality of the content uploaded on YouTube must be evaluated properly to ensure that accurate and reliable information is being disseminated to caregivers and children with CP. Consequently, this study aims to analyze YouTube-based therapeutic content for children with CP by focusing on the qualitative and quantitative aspects of the videos and therapeutic programs.

## 2. Materials and Methods

### 2.1. Study Design and Video Selection Strategy

A keyword search was conducted on 12 January 2024; specific terms related to neuromotor therapy or exercise for CP were used, including “cerebral palsy + exercise”, “cerebral palsy + physical therapy”, “cerebral palsy + physiotherapy”, “cerebral palsy + rehabilitation”, “cerebral palsy + sensorimotor therapy + rood”, “cerebral palsy + vojta therapy”, “cerebral palsy + sensory integration + ayres”, “cerebral palsy + patterning therapy + doman-delacato”, and “cerebral palsy + neurodevelopmental treatment + bobath”. A Python-based crawling technique was employed to search all relevant videos on YouTube.

**Figure 1:**
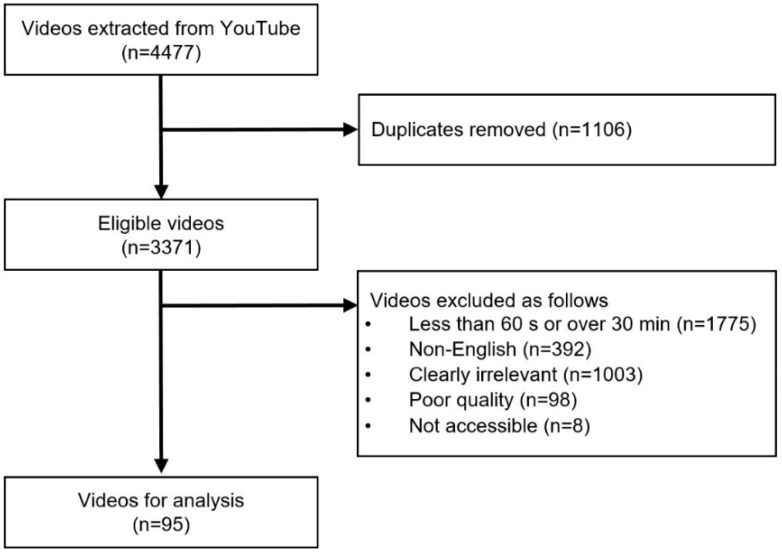
Flowchart demonstrating the video selection process.

The exclusion criteria included videos that were duplicates, unavailable, not in English, and irrelevant, for example, those containing game suggestions or promotional materials for a clinic. Additionally, videos lacking verbal instructions or explanations in scripts were deemed to be of poor quality and excluded, along with those with a duration of less than 60 s or longer than 30 min. Figure 1 presents a flowchart outlining the video selection process.

Our research exclusively utilized publicly available online videos without direct interaction with humans or animals, and personally identifiable information was not collected or used; hence, ethical committee approval was not required. 

### 2.2. Data Acquisition and Classification

Basic characteristics of the videos, including the title, uniform resource locator, upload date, playtime, and information about the uploader and therapeutic intervention, were collected. Uploaders were divided into two categories: health professionals (e.g., physical therapists) and non-professionals (affiliations unknown). Types of neuromotor therapy were categorized as follows: “Bobath”, “sensorimotor approach to treatment (Rood)”, “sensory integration approach (Ayres)”, “Vojta approach”, and “patterning therapy (Doman-Delacato)”. These categories were established based on recognized neuromotor therapy approaches that have been extensively studied for their effectiveness in treating CP within academic research [13,14]. Types of exercise were categorized based on the guidelines of the American College of Sports Medicine (ACSM) for chronic disability [15] as follows: “aerobic”, “strengthening”, “flexibility”, “warm-up and cool-down”, and “physical functioning exercise”. For classifications of neuromotor therapy and exercises, if two or more of the criteria were satisfied, the video was classified as a “mixed type”. If no criteria were satisfied, it was categorized as an “unclassified type”.

Two researchers (YD and YO) evaluated all the videos included in this study according to three domains: the popularity of the video, the quality of video content, and the quality of therapeutic programs. In cases of discrepancies between the two evaluators, a third researcher (JH) intervened, and the differences were discussed until consensus was attained. All researchers had more than five years of experience in physical therapy or pediatric rehabilitation. 

#### 2.2.1. Popularity-Related Parameters of Videos

Popularity-related information, such as the number of likes, dislikes, and total views, was collected. Additionally, the view ratio and video power index (VPI), representing a video’s popularity, were calculated using the following equations [16]:

VPI = View ratio* × Like ratio**(1)

View ratio* = Total number of views/Number of days since uploaded(2)

Like ratio** = (Total number of likes × 100)/(Total number of likes + Total number of dislikes)(3)

#### 2.2.2. Assessment Tools for Quality of Video Content

A modified DISCERN (mDISCERN) tool and Global Quality Scale (GQS) were used to assess the quality of the informational videos. Both tools are commonly used to evaluate the content quality of YouTube videos [8]. The mDISCERN tool ranges from 0 to 5 points and comprises five items: “clarity of aim”, “reliability of information sources”, “balanced and unbiased information”, “listing of additional information”, and “mention of uncertainty or controversy”. Each criterion was evaluated by not only the video itself but also the video title and script. These were rated on a 2-point scale in this study to evaluate the reliability of video content; 1 point was given when the video fulfilled the criteria of each item, with a higher score indicating better reliability. A score of 3 or higher out of a maximum of 5 points indicates reliability, while a score below indicates unreliability [17,18]. The GQS ranges from 1 to 5 points, designed to rate the flow and quality of online information [8,19]. “Poor quality & flow” corresponds to a score of 1 point, while “excellent quality & flow” corresponds to a score of 5 points, indicating higher scores for higher-quality content. The information quality item was assessed based on whether the content clearly stated its purpose, provided appropriate content for its purpose, and included additional information. The engagement quality item assessed whether there were initial hooks in the content, an introduction that addressed the topic and summarized it, explanations about the content, and devices to enhance adherence toward the end of the video. Scores from 4 to 5 on the GQS were classified as high quality, a score of 3 as moderate, and scores from 1 to 2 as low quality [17]. 

#### 2.2.3. Assessment Tools for Quality of Therapeutic Programs

The International Consensus on Therapeutic Exercise and Training (i-CONTENT) tool, Consensus on Therapeutic Exercise Training (CONTENT) scale, and Consensus on Exercise Reporting Template (CERT) were used to analyze the quality of therapeutic interventions in the videos [20,21,22]. The i-CONTENT tool comprises seven items: “patient selection”, “dosage of the training program”, “type of training program”, “presence of qualified supervisor”, “type and timing of outcome assessment”, “safety of the training program”, and “adherence to the training program”. A rating scheme developed by Voorn et al. [21] was used for the evaluation, grouping videos into three categories: low, some, or high risk of ineffectiveness. 

The CONTENT scale comprises five critical areas: “patient eligibility”, “competencies and setting”, “rationale”, “content”, and “adherence” [20,23]. Each area was evaluated using a binary system, assigning 1 point for “yes” and 0 points for “no”. A program with 6 points or higher out of 9 was considered to have high therapeutic quality [20,23]. 

The CERT comprises 19 items in an extended version of the Template for Intervention Description and Replication checklist, used to examine the completeness of exercise descriptions. Each item is rated on a binary scale of 0 and 1, similar to the CONTENT scoring method. A higher score indicates that the content includes more comprehensive information [20,22,24]. 

### 2.3. Statistical Analysis

All statistical analyses were conducted using the Statistical Product and Service Solutions software (IBM Corp, Armonk, NY, USA) version 23.0. Continuous variables are presented as mean ± standard deviation and median with interquartile range, while categorical variables are reported as frequencies and percentages. A chi-squared test assessed the association between quality analysis tools and specific subgroups based on uploader types and the classification of therapy type. To analyze the consistency between the evaluation results of the quality assessment tools and popularity-related scales, as well as between the outcomes of the qualitative analysis for video content and treatment programs, Spearman’s Rank Correlation was used. The scale of correlation coefficients was based on previous studies [25]. Statistical significance was set at *p* < 0.05.

## 3. Results

### 3.1. General Characteristics and Quantitative Analysis of Video Content

**Figure 2:**
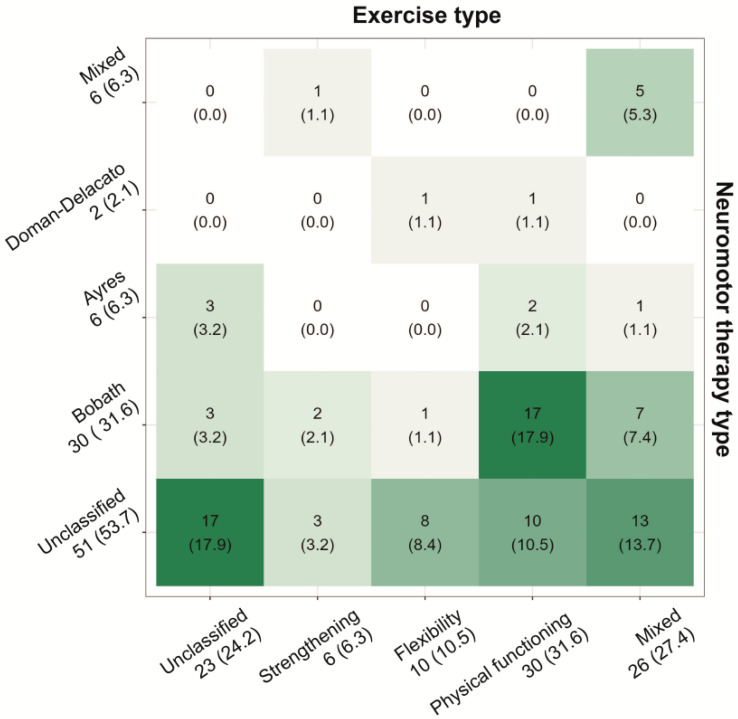
Confusion matrix between types of neuromotor treatment modalities and exercises. The higher the percentage the occupies, the darker the shading used for visualization. Therapeutic types, such as Rood, Vojta approach, and aerobic, warm-up, and cool-down exercise, which were not represented in any of the videos, were excluded from the visualization.

A total of 4477 videos were collected using the Python-based crawling technique. Among them, 95 videos that met the inclusion criteria were evaluated in this study. The majority of videos were uploaded by health professionals (73, 76.8%). Regarding types of neuromotor therapy, most videos were categorized as “unclassified” (51, 53.7%). Additionally, over 30% of the total videos (30, 31.6%) included the Bobath approach, while the Ayres (6, 6.3%), Doman-Delacato (2, 2.1%), and Rood and Vojta approaches (0, 0.0%) were represented in less than 10%. Concerning exercise, the “unclassified type” accounted for only 24.2% of the videos, contrasting with the results for neuromotor therapy. The most common type was physical functioning exercise (30, 31.6%), followed by the mixed type (26, 27.5%), flexibility (10, 10.5%), strengthening (6, 6.4%), and warm-up and cool-down exercises (0, 0.0%). An overview of the general features of the included videos is provided in Table 1. In the confusion matrix between the two classification methods, the most frequent instances were videos that could not be classified by either method or those categorized under the Bobath type as physical functioning exercise (17, 17.9%) (Figure 2).

### 3.2. Analysis of Qualitative Assessment for Video Content and Treatment Program

**Figure 3:**
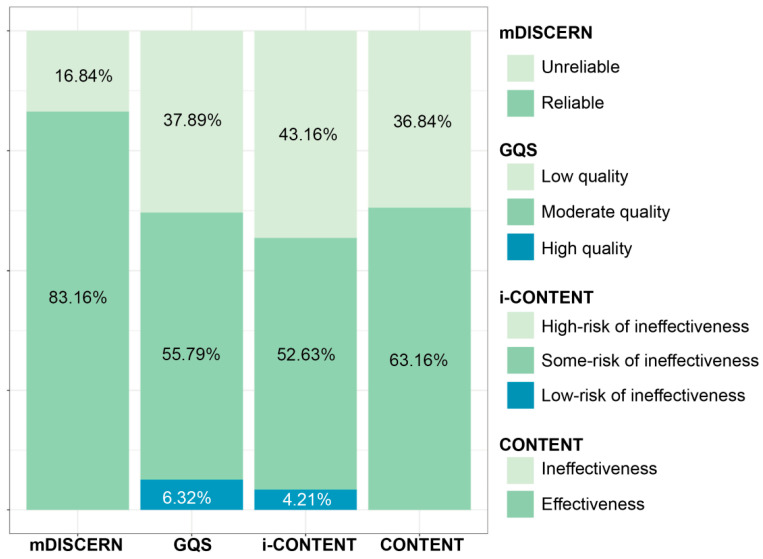
Classification of assessment tools for video content and therapeutic programs. Abbreviations: mDISCERN, modified DISCERN; GQS, Global Quality Scale; i-CONTENT, International Consensus on Therapeutic Exercise and Training; CONTENT, Consensus on Therapeutic Exercise Training.

Table 2 presents the results of the evaluation of video content quality using mDISCERN and GQS. The mean mDISCERN score was 3.77 ± 0.12 out of 5, and 83.16% (n = 79) of the videos were classified as having reliable content (Figure 3). Analysis of each item revealed that over 75% of the videos demonstrated “clarity of aim”, “balanced and unbiased information”, “listing of additional information”, and “reliability of sources cited”. However, only 46.3% made “mention of uncertainty or controversy”.

The mean score of GQS was 2.64 ± 0.67 out of 5. Most content was evaluated as being of “moderate quality and flow” (55.8%) and “generally poor quality and flow” (33.7%). This indicates that, while most content included some information, it often omitted crucial details or provided crucial information on some aspects but not on others. No content met the criterion for having “excellent quality and flow” (Table 2). Based on GQS classification scores, 37.9% of the videos were categorized as low quality, 55.8% as moderate quality, and 6.3% as high quality (Figure 3).

Table 3 presents the results of the therapeutic quality of programs based on i-CONTENT, CONTENT, and CERT. The mean score of i-CONTENT was 3.88 ± 0.11 out of 7. According to i-CONTENT, 43.16% of the videos were at high risk of ineffectiveness, while 52.63% and 4.21% had some or a low risk of ineffectiveness, respectively (Figure 3). Specifically, only two videos (2.1%) satisfied the item “type and timing of outcome assessment”. Furthermore, items related to safety (31, 32.6%) and dosage (34, 35.8%) of the program scored less than half the satisfaction rate, contrasting with other items related to patient selection, types of programs, and adherence.

The mean score of CONTENT was 6.00 ± 0.20 out of 9, and 63.16% (n = 60) of videos were classified as effective in terms of content quality (Figure 3). Most videos (96.8%) obtained scores for the item “competencies and setting”. However, for content-related items, less than 50% met the criteria, indicating an overall low level of fulfillment.

The mean score of CERT was 7.37 ± 0.33 out of 19. For each item, over 90% of videos met the criterion of providing a “detailed explanation of each exercise to facilitate replication”. However, less than half of the videos obtained scores for exercise individualization (Items 3, 4, 6, 7, 9, 13, and 14), adverse effects (Item 11), set-up (Items 12 and 15), and adherence monitoring methods during the program (Item 16).

### 3.3. Relationship between the Qualitative Assessments of Video Content and Uploader Type

Of the 95 videos analyzed, 73 were uploaded by health professionals and 22 by non-professionals. Table 4 presents the results of video content quality evaluation using each tool for each type of uploader. Significant relationships were observed between the results of mDISCERN and GQS for the uploader types (*p* < 0.001, *p* = 0.001, respectively). Videos uploaded by health professionals exhibited higher-quality video content. However, no relationships were observed between the types of uploaders and the results of the i-CONTENT and CONTENT tools.

### 3.4. Relationship between Quality Assessment and Ability to Classify Types of Therapeutic Interventions

Table 5 illustrates the association between the quality analysis and classifiable video content based on types of neuromotor therapy and exercise. All videos meeting classification criteria were categorized as “classified”. When divided by types of neuromotor therapy, no significant qualitative difference was observed between the two groups. However, significant qualitative differences in video and therapeutic program quality were observed when divided by exercise type.

### 3.5. Correlation between Qualitative and Quantitative Assessments of Video Content

A comparison of popularity-related parameters, including VPI and view ratio, with the quality analysis tools of video content and therapeutic intervention, indicated no statistically significant correlation between all quality assessment tools and the popularity-related index (Table 6).

### 3.6. Correlation between Qualitative Assessment Tools

**Figure 4:**
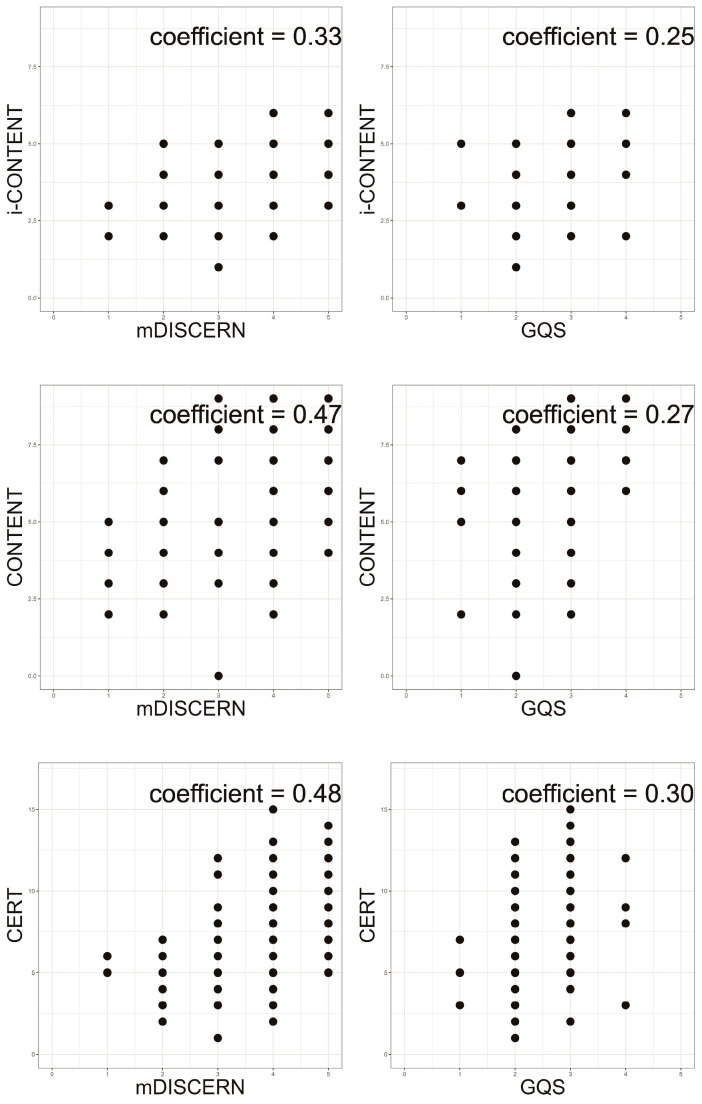
Correlation plots for the qualitative assessments of video content and therapeutic programs. Abbreviations: mDISCERN, modified DISCERN; GQS, Global Quality Scale; i-CONTENT, International Consensus on Therapeutic Exercise and Training; CONTENT, Consensus on Therapeutic Exercise Training; CERT, Consensus on Exercising Reporting Template.

Spearman rank correlation analyses revealed positive correlations between the evaluation tools (Table 7, Figure 4). mDISCERN exhibited a moderate correlation between mDISCERN and CONTENT (rho = 0.466, *p* < 0.001) and CERT (rho = 0.483, *p* < 0.001) and a weak correlation with i-CONTENT (rho = 0.334, *p* < 0.001). Additionally, a weak correlation was found between GQS and the other tools (i-CONTENT: rho = 0.249, *p* = 0.015; CONTENT: rho = 0.270, *p* = 0.008; CERT: rho = 0.302, *p* = 0.003).

## 4. Discussion

To our knowledge, this study is the first to quantitatively and qualitatively analyze web-based YouTube videos focused on children with CP. This study aims to conduct a qualitative analysis of the delivery of therapeutic interventions through video content, not a comparative study of the therapeutic effectiveness between them. 

Regarding the categorization by exercise type, physical functioning and mixed exercises constituted the majority of videos, with no content related to aerobic, warm-up, and cool-down exercises, which are emphasized in the ACSM guidelines for individuals with CP [15]. Future content development should prioritize these exercises and their safe application to pathological conditions. Regarding neuromotor therapy types, a significant portion of videos fell under the “unclassified” category, indicating a lack of a widespread introduction to various neuromotor therapeutic modalities on current online platforms. As home-based exercise gains importance, there is a critical need for online platforms to develop videos that introduce these therapies.

Furthermore, in the confusion matrix comparing the two classification methods, 17.9% of the videos focused on Bobath therapy with physical functioning exercises, representing the highest proportion observed in this study. The significant presence of videos covering both the Bobath approach and physical functioning exercises could be interpreted as reflecting characteristics of this neuromotor therapy, activity, and participation-based interventions, which is one of the main components of the Bobath Clinical Reasoning Framework (BCRF) model aligning with the International Classification of Functioning (ICF) categories [26]. Meanwhile, the substantial number of videos that do not fit clearly into either classification (17.9% of all videos) underscores the necessity for higher-quality educational videos that differentiate between various exercise and therapeutic techniques.

A qualitative evaluation of the videos indicates that more than half were of moderate or higher quality. Regarding specific items, most criteria in the mDISCERN tool achieved a satisfaction rate of over 75%. This suggests that the majority of videos provided detailed descriptions of actions, effectively conveying the video’s information and purpose through visuals or video footage. However, only 46.3% of videos satisfied the criterion for the item “mention of uncertainty or controversy”. This indicates a tendency for videos to focus on effectiveness rather than potential uncertainties or adverse effects. Additionally, the results from the GQS, which assesses information quality and flow, show that none of the videos met the criteria for “excellent quality and flow”. This is primarily because few videos met the “good to excellent” standards for both criteria, highlighting a limitation in providing detailed “exercise information” on YouTube. This reflects a common challenge with YouTube content, wherein the need for quick information delivery to maintain viewer engagement can affect the depth and structure of educational content [27]. 

The qualitative assessment of therapeutic programs classified 43.16% of videos using i-CONTENT and 36.84% using CONTENT as ineffective programs. The videos primarily focused on explaining the type of exercise introduced but often lacked other essential elements of the frequency, intensity, time, and type (FITT) principle [28], crucial for prescribing effective exercise programs. Specifically, elements such as “dosage of the training program” in i-CONTENT, content-related items in CONTENT, and Items 3, 4, 6, 7, 9, 13, and 14 in CERT are integral to constructing the FITT model. Additionally, aspects related to outcome measurement during or after therapeutic intervention (e.g., “type and timing of outcome assessment” in i-CONTENT and Items 16a and 16b in CERT), descriptions of adverse events (e.g., “safety of the training program” in i-CONTENT and Question 11 in CERT), and basic exercise setup (e.g., Questions 12 and 15 on CERT), such as precautions and subject posture, were significantly lacking.

In the comparative quality analysis between videos uploaded by health professionals and those by non-professionals, although the majority of videos were produced by health professionals, a significant difference was observed only in video quality. No significant difference was found in therapeutic content quality. This suggests that even videos produced by health professionals did not consistently provide disease-specific content for therapeutic interventions targeting children with CP. Future video production efforts should prioritize filling this gap. 

Furthermore, videos that could be classified by exercise type showed significant differences in therapeutic quality evaluation compared with unclassifiable videos. In contrast, there was no statistically significant difference in quality between groups based on the classification of neuromotor therapy. There are many doubts about the clinical efficacy of neuromotor treatment. This is because the decision-making process in neuromotor therapy, which involves non-linear, and thinking-based clinical reasoning, poses significant constraints in proposing standardized treatment methods [29]. To overcome the shortcomings of these conventional neuromotor therapies, child-active or functional therapy approaches that involve actively practicing real-life tasks, especially in real-life environments [30,31,32,33], have been proposed. To establish clinical evidence for these therapeutic techniques, it is essential to develop tailored strategies and ensure the articulate delivery of these therapeutic techniques. 

No correlations were found between the results of the quality evaluation tools and popularity-related parameters. This indicates that the degree of video popularity does not guarantee the qualitative aspect of therapeutic educational videos. However, a statistically significant correlation was found between the therapeutic quality evaluation tools. Specifically, mDISCERN demonstrated a more significant correlation with the therapeutic quality evaluation tools compared with the GQS, potentially influenced by the relatively large standard deviation of the GQS scores.

This study had several limitations. First, the sample size was relatively small to comprehensively analyze video quality. Second, the treatment program assessment tools used were originally designed for evaluating in-person treatment programs, which may not fully capture the nuances of assessing online treatment programs. There is a clear need for the development of new evaluation tools tailored for assessing video-based programs that comprehensively evaluate both video content and treatment programs beyond the constraints of traditional in-person evaluations. Third, significant differences in the proportions between groups based on types of neuromotor therapy and exercise made statistical comparisons challenging. Therefore, future studies should aim to compare treatment modalities more effectively. Fourth, this study may have focused exclusively on well-known neuromotor interventions and may not have included other approaches, such as the functional therapy approach. Future research could consider including a variety of approaches to broaden the scope of the investigation. Finally, this study is limited to a qualitative analysis conducted by researchers. Future research may focus on practical clinical application, for example, by conducting a satisfaction analysis with actual users, like caregivers, patients, and clinicians, or a prospective study on the clinical effects by applying information acquired through video content.

## 5. Conclusions

YouTube-based therapeutic videos for children with CP generally exhibit reliability in video content and effectiveness in therapeutic programming. However, no correlations were found between video popularity and quality. Qualitative analysis revealed a significant omission regarding the mention of uncertainty in the treatment principles within video content. Therapeutically, there was a notable absence of detailed treatment descriptions encompassing aspects such as intensity, frequency, timing, setting, outcome measurement during and post-treatment, and safety considerations. This deficiency was consistent regardless of the uploader’s expertise level and the classification of neuromotor therapy type. These results imply that YouTube-based content for CP children still has significant limitations in how caregivers, patients, and clinicians, as substantive viewers, can acquire tailored information and apply practical information to their exercise and treatment programs. In the future, further refinement of the content for CP children is essential to ensure the delivery of accurate and reliable information on exercise and neuromotor therapy. 

## Figures and Tables

**Table 1:** General characteristics and quantitative analysis of the videos.

General Characteristics of Videos	Value
Total videos (n)	95
Health professional uploader (n [%])	73 (76.8)
Neuromotor therapy type (n [%])	
Bobath	30 (31.6)
Rood	0 (0.0)
Ayres	6 (6.3)
Vojta approach	0 (0.0)
Doman-Delacato	2 (2.1)
Mixed	6 (6.3)
Unclassified	51 (53.7)
Exercise type (n [%])	
Aerobic	0 (0.0)
Strengthening	6 (6.3)
Flexibility	10 (10.5)
Warm-up and cool-down	0 (0.0)
Physical functioning	30 (31.6)
Mixed	26 (27.4)
Unclassified	23 (24.2)
Views (Median [IQR])	6571.00 (2514.0–36,692.00)
Likes (Median [IQR])	82.00 (25.0–322.00)
Dislikes (Median [IQR])	0.00 (0.0–8.00)
VPI (Median [IQR])	6.08 (2.15–25.02)

Abbreviations: IQR, interquartile range; VPI, video power index.

**Table 2:** Results of the quality assessment of video content.

Evaluation Tool	Value
mDISCERN total (Mean ± SD)	3.768 ± 0.117
Clarity of aim (n [%])	85 (89.5)
Reliability of information source (n [%])	73 (76.8)
Balanced and unbiased information (n [%])	78 (82.1)
Listing of additional information (n [%])	78 (82.1)
Mention of uncertainty or controversy (n [%])	44 (46.3)
GQS total (Mean ± SD)	2.64 ± 0.667
Poor quality and flow (n [%])	4 (4.2)
Generally poor quality and flow (n [%])	32 (33.7)
Moderate quality and flow (n [%])	53 (55.8)
Good quality and flow (n [%])	6 (6.3)
Excellent quality and flow (n [%])	0 (0.0)

Abbreviations: mDISCERN, modified DISCERN; SD, standard deviation; GQS, Global Quality Scale.

**Table 3:** Results of the quality assessment for therapeutic programs.

Evaluation Tool	Value
i-CONTENT total (Mean ± SD)	3.884 ± 0.1108
Patient selection (n [%])	66 (69.5)
Dosage of training program (n [%])	34 (35.8)
Type of training program (n [%])	90 (94.7)
Presence of qualified supervisor (n [%])	84 (88.4)
Type and timing of outcome assessment (n [%])	2 (2.1)
Safety of training program (n [%])	31 (32.6)
Adherence to training program (n [%])	64 (67.4)
CONTENT total (Mean ± SD)	6.0 ± 0.1951
Patient eligibility (n [%])	
Description of patient selection	57 (60)
Adequacy of patient selection	86 (90.5)
Competences and setting (n [%])	
Determination and adequacy of eligibility criteria for therapist and setting	92 (96.8)
Rationale (n [%])	
Basis of therapeutic exercise on a priori aims and intentions	84 (88.4)
Description and plausibility of the rationale for the content and intensity of the therapeutic exercise	69 (72.6)
Content (n [%])	
Description of the intensity of the therapeutic exercise	33 (34.7)
Monitoring and adjustment of the therapeutic exercise when necessary	44 (46.3)
Personalization and contextualization of the therapeutic exercise to individual participants	44 (46.3)
Adherence (n [%])	
Determination and acceptability of adherence to the therapeutic exercise	65 (68.4)
CERT total (Mean ± SD)	7.379 ± 0.3313
1. Detailed explanation of type of exercise equipment (n [%])	54 (56.8)
2. Detailed overview of the exercise instructor’s qualifications, teaching/supervising expertise, and training (n [%])	65 (68.4)
3. Description of whether exercises are conducted individually or in a group (n [%])	37 (38.9)
4. Description of whether exercises are supervised or unsupervised, and their delivery method (n [%])	24 (25.3)
5. Detailed explanation of how exercise adherence is measured and reported (n [%])	63 (66.3)
6. Detailed explanation of motivation strategies (n [%])	39 (41.1)
7a. Detailed explanation of the decision rule(s) used to determine exercise progression (n [%])	12 (12.6)
7b. Detailed explanation of progression of the exercise program (n [%])	11 (11.6)
8. Detailed explanation of each exercise to facilitate replication (n [%])	90 (94.7)
9. Detailed explanation of any home program component (n [%])	47 (49.5)
10. Explanation of the presence of any non-exercise components (n [%])	55 (57.9)
11. Description of the type and frequency of adverse events observed during exercise (n [%])	7 (7.4)
12. Description of the setting in which the exercises are conducted (n [%])	9 (9.5)
13. Comprehensive description of the exercise intervention, including various aspects such as the number of exercise repetitions, sets per session, session duration, program duration, etc. (n [%])	33 (34.7)
14a. Explanation of whether the exercises are generic (one size fits all) or tailored (n [%])	42 (44.2)
14b. Description of how exercises are tailored to the individual (n [%])	33 (34.7)
15. Description of the criteria used to establish the initial level for individuals starting an exercise program (n [%])	11 (11.6)
16a. Explanation of how adherence or fidelity is assessed/measured (n [%])	35 (36.8)
16b. Explanation of the extent to which the intervention was delivered as planned (n [%])	34 (35.8)

Abbreviation: i-CONTENT, International Consensus on Therapeutic Exercise and Training; SD, standard deviation; CONTENT, Consensus on Therapeutic Exercise Training; CERT, Consensus on Exercising Reporting Template.

**Table 4:** Results of the differences between groups by uploader expertise and quality assessment tool.

Qualitative Evaluation Domain	Qualitative Evaluation Tool	Classification	Uploader			χ^2^ (*p*)
			Non-Professional	Health Professional	Total	
Quality of video content	mDISCERN (n [%])	Unreliable	11 (68.75)	5 (31.25)	16 (100)	22.475 (<0.001)
		Reliable	11 (13.92)	68 (86.08)	79 (100)	
		Total	22 (23.16)	73 (76.84)	95 (100)	
	GQS (n [%])	Low quality	16 (44.44)	20 (55.56)	36 (100)	15.148 (0.001)
		Moderate quality	6 (11.32)	47 (88.68)	53 (100)	
		High quality	0 (0.00)	6 (100)	6 (100)	
		Total	22 (23.16)	73 (76.84)	95 (100)	
Quality of treatment programs	i-CONTENT (n [%])	High risk of ineffectiveness	13 (31.71)	28 (68.29)	41 (100)	3.637 (0.162)
		Some risk of ineffectiveness	9 (18.00)	41 (82.00)	50 (100)	
		Low risk of ineffectiveness	0 (0.00)	4 (100)	4 (100)	
		Total	22 (23.16)	73 (76.84)	95 (100)	
	CONTENT (n [%])	Ineffectiveness	11 (31.43)	24 (68.57)	35 (100)	2.130 (0.144)
		Effectiveness	11 (18.33)	49 (81.67)	60 (100)	
		Total	22 (23.16)	73 (76.84)	95 (100)	

Abbreviations: mDISCERN, modified DISCERN; GQS, Global Quality Scale; i-CONTENT, International Consensus on Therapeutic Exercise and Training; CONTENT, Consensus on Therapeutic Exercise Training.

**Table 5:** Results of differences between quality assessment tools and classification of therapeutic type.

Qualitative Evaluation Tool	Classification	Neuromotor Therapy			χ^2^ (*p*)	Exercise			χ^2^ (*p*)
		Unclassified	Classified	Total		Unclassified	Classified	Total	
i-CONTENT (n [%])	High risk of ineffectiveness	25 (61.0)	16 (39.0)	41 (100)	2.473 (0.290)	25 (61.0)	16 (39.0)	41 (100)	6.264 (0.044)
	Some risk of ineffectiveness	25 (50.0)	25 (50.0)	50 (100)		25 (50.0)	25 (50.0)	50 (100)	
	Low risk of ineffectiveness	1 (25.0)	3 (75.0)	4 (100)		1 (25.0)	3 (75.0)	4 (100)	
	Total	51 (53.7)	44 (46.3)	95 (100)		51 (53.7)	44 (46.3)	95 (100)	
CONTENT (n [%])	Ineffectiveness	19 (54.3)	16 (45.7)	35 (100)	0.008 (0.928)	19 (54.3)	16 (45.7)	35 (100)	10.501 (0.001)
	Effectiveness	32 (53.3)	28 (46.7)	60 (100)		32 (53.3)	28 (46.7)	60 (100)	
	Total	51 (53.7)	44 (46.3)	95 (100)		51 (53.7)	44 (46.3)	95 (100)	

Abbreviations: i-CONTENT, International Consensus on Therapeutic Exercise and Training; CONTENT, Consensus on Therapeutic Exercise Training.

**Table 6:** Correlation results of quantitative and qualitative assessments of video content and therapeutic programs.

	Quality of Video Content		Quality of Therapeutic Program		
	mDISCERN	GQS	i-CONTENT	CONTENT	CERT
VPI	0.190 (0.068)	0.191 (0.067)	0.112 (0.287)	0.009 (0.934)	−0.034 (0.748)

Abbreviations: VPI, video power index; mDISCERN, modified DISCERN; GQS, Global Quality Scale; i-CONTENT, International Consensus on Therapeutic Exercise and Training; CONTENT, Consensus on Therapeutic Exercise Training; CERT, Consensus on Exercising Reporting Template.

**Table 7:** Correlation results for the qualitative assessments of video content and therapeutic programs.

	i-CONTENT	CONTENT	CERT
mDISCERN	0.334 (0.001)	0.466 (<0.001)	0.483 (<0.001)
GQS	0.249 (0.015)	0.270 (0.008)	0.302 (0.003)

Abbreviations: mDISCERN, modified DISCERN; GQS, Global Quality Scale, i-CONTENT, International Consensus on Therapeutic Exercise and Training; CONTENT, Consensus on Therapeutic Exercise Training; CERT, Consensus on Exercising Reporting Template.

## Data Availability

The data presented in this study are available on request from the corresponding author. The data are not publicly available due to restrictions e.g., privacy or ethics.
